# A novel and alternative therapy for persistent allergic rhinitis via intranasal acupuncture: a randomized controlled trial

**DOI:** 10.1007/s00405-022-07793-x

**Published:** 2023-01-09

**Authors:** Li-li Liu, Zheng Gong, Lei Tang, Zhan-feng Yan

**Affiliations:** 1 Department of Otorhinolaryngology, Dongzhimen Hospital, Beijing University of Chinese Medicine, 1 No. 5, Haiyuncang Hutong, Dongcheng District, Beijing, 100020 People’s Republic of China; 2 The Third Hospital of Beijing Chaoyang District, No. A5, Shuangqiao South Road, Chaoyang District, Beijing, 100025 People’s Republic of China

**Keywords:** Persistent allergic rhinitis (PAR), Intranasal acupuncture, Olfactory function, Nasal nerve adjustment

## Abstract

**Background:**

Acupuncture is used to treat allergic rhinitis (AR) in traditional Chinese medicine, and the ST2 and ST36 acupoints are generally selected in clinical practice. We report a new intranasal acupuncture method at the Neiyingxiang (EX-HN9) and Biqiu points for the treatment of persistent AR (PAR). Here, the efficacy and safety of this method were evaluated.

**Methods:**

A total of 120 patients diagnosed with PAR were randomly allocated (2:1 ratio) to intranasal acupuncture or Western medicine groups, the basic principle of random grouping is SAS random grouping method. The applicator held a nasal endoscope and a 0.30 × 75 mm filiform needle in their left and right hands, respectively. When aiming at the Neiyingxiang or Biqiu point, the applicator quickly inserted the needle to a 20-mm depth as parallel as possible to the inferior turbinate or middle turbinate, without special reinforcing and reducing techniques (the needle remained for 20 min). The intranasal acupuncture groups received acupuncture treatment three times per week for 2 weeks. The Western medicine group was treated with budesonide nasal spray (two sprays/nostril, twice/day) and loratadine (one tablet/night) for 2 weeks. Visual analog scale (VAS) scores were the primary outcome. Quality of life, medication dosages and adverse events were secondary outcomes measured using the Rhinoconjunctivitis Quality-of-Life Questionnaire (RQLQ). Confidence assessments were performed to evaluate data from the treatment and follow-up periods.

**Results:**

The results were as follows: (1) VAS and RQLQ scores were significantly lower in the intranasal acupuncture group than in the Western medicine group on day 1 (i.e., first treatment) (*P* < 0.05; 95% CI − 13.1 to − 9.6 VAS points) (*P* < 0.05; 95% CI − 20.27 to − 12.28 RQLQ points). Overall symptoms (95% CI − 2.86 to − 1.86 points), nasal obstruction (95% CI − 6.33 to − 5.36 points), olfactory function (95% CI − 2.91 to − 1.75 points), sleep (95% CI − 5.05 to − 3.57 points), actual problems (95% CI − 2.03 to − 0.06 points), nasal symptoms (95% CI − 6.62 to − 4.5 points), and emotional problems (95% CI − 5.05 to − 3.5 points) were significantly improved. (2) VAS and RQLQ scores in the two groups were significantly improved at week 2; however, there were no significant group differences in the VAS (*P* > 0.05; 95% CI − 1.21 to − 1.38 points) and RQLQ (*P* > 0.05; 95% CI − 0.33 to − 3.46 points) scores. Olfactory function symptoms were significantly improved (95% CI − 1.58 to − 0.21 points). (3) During the follow-up period, there was a significant difference between the two groups (*P* < 0.05) with higher RQLQ and VAS scores in the intranasal acupuncture group than in the Western medicine group. VAS scores on rhinobyon symptoms, nasal itch, rhinorrhea and olfactory function and RQLQ scores for activities, non-nasal/eye symptoms, actual problems, nasal symptoms, and eye symptoms were significantly improved. (4) No adverse events were observed in either group during treatment.

**Conclusions:**

Intranasal acupuncture has good efficacy and safety in the treatment of PAR. Moreover, VAS and RQLQ scores were much lower in the intranasal acupuncture group than in the Western medicine group, and acupuncture had an immediate impact, especially for improving nasal congestion, olfactory function and sleep.

## Background

Allergic rhinitis (AR) is a chronic respiratory allergic disease mediated by IgE that is characterized by the clinical symptoms of sneezing, itching, rhinorrhea and nasal blockage. It affects 10–40% of the population, approximately one billion people worldwide [1]. The prevalence of AR in European adults was reported to range from 17% to 28.5% [2]. Typical incidence reports are between 10% and 30% of children and adults in the United States and other developed nations [3]. Persistent allergic rhinitis (PAR) is a common type of AR with symptoms lasting more than 4 days a week for 4 weeks [4]. Recently, the incidence of PAR and the severe chronic systemic diseases caused by it have been on the rise and show a trend of impacting younger individuals. It is associated with a substantial health and psychological burden in patients due to its etiologically complex, prolonged disease course and high incidence.

Currently, conventional medical treatment can effectively relieve AR symptoms. However, there is still poor clinical efficacy in some patients and adverse effects of these medications. Although no comprehensive assessment of their antiallergic–rhinitis mechanisms has been published, and scientific results have yet been undertaken, traditional Chinese medicine (TCM) herbs are beneficial against AR [5]. Treatment that combines complementary and alternative medicine (CAM) improves clinical efficacy and reduces the incidence of adverse reactions [6].

Acupuncture is a widely used and relatively safe nonpharmacological treatment for some conditions. It is gradually becoming an important complementary and alternative strategy for the treatment of AR [7]. In clinical practice, more than 25% of AR patients receive TCM treatment, among which 17% receive acupuncture therapy [8]. Acupuncture treatment has become a safe and effective option to relieve AR symptoms and improve quality of life [8, 9]. Meanwhile, acupuncture is now listed in the AR guidelines in the United States [10].

**Fig. 1:**
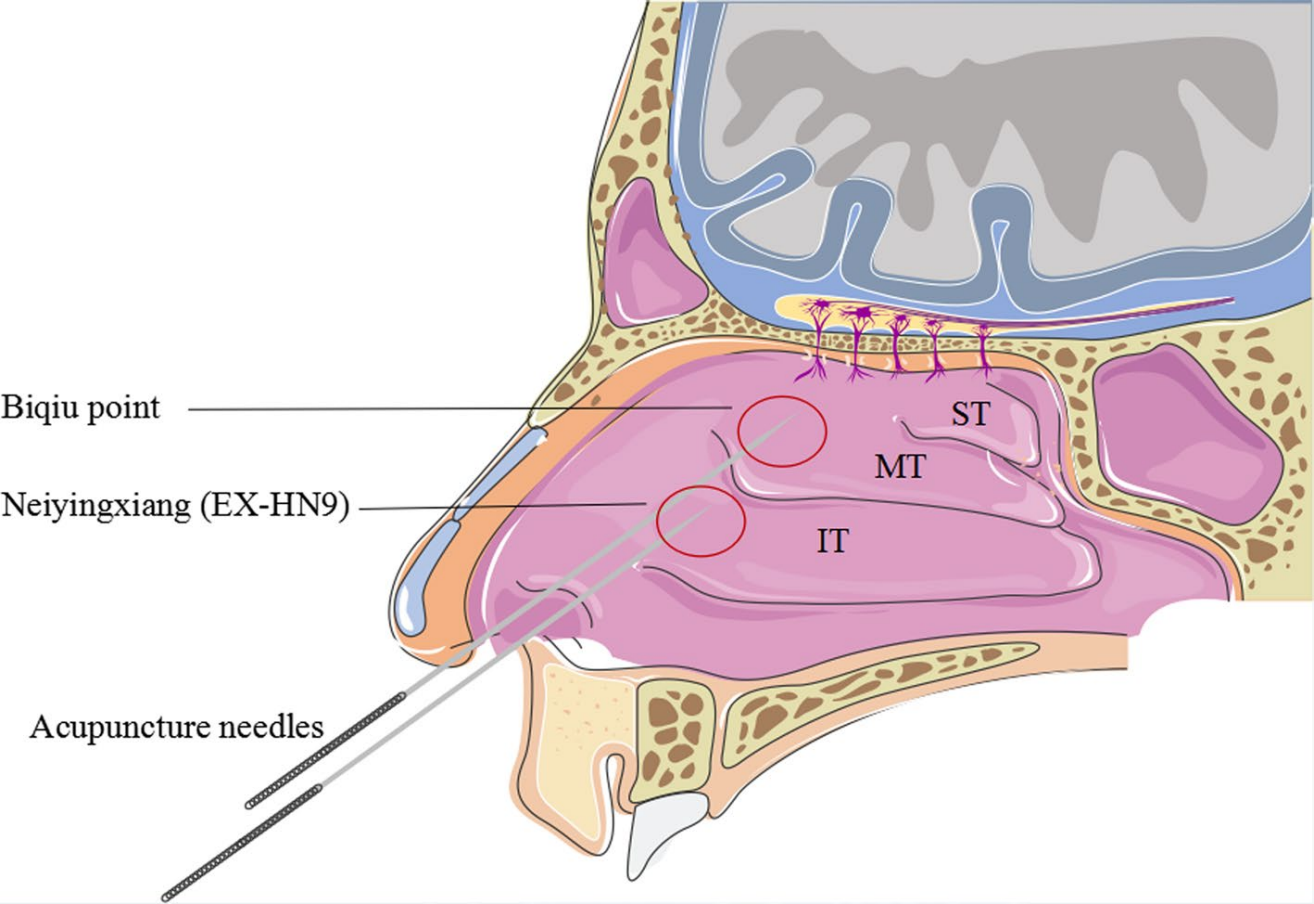
Schematic diagram of intranasal acupuncture (When aiming at the Neiyingxiang or Biqiu point, the applicator quickly inserted the needle to a 20-mm depth as parallel as possible to the inferior turbinate or middle turbinate, without special reinforcing and reducing techniques (the needle remained for 20 min). *ST* superior turbinate, *MT* middle turbinate, *IT* inferior turbinate

We have applied a novel and alternative therapy for PAR via intranasal acupuncture (IA) in the clinic and achieved good effects. IA is a treatment involving the needling of specific areas (Neiyingxiang (EX-HN9) and Biqiu points) in the nasal cavity (Fig. 1). Neiyingxiang (EX-HN9) is located inside and above the nostril and is in the mucosal area at the junction of the inferior turbinate root and the lateral wall of the nasal cavity. The Biqiu point is located in the front of the middle turbinate of the lateral wall of the nasal cavity. The areas with the Neiyingxiang (EX-HN9) and Biqiu points have abundant nerve endings. The curative effect of acupuncture on PAR is through the modulation of the sympathetic and parasympathetic nerve interactions to produce anti-inflammatory effects that directly inhibit the development of AR and thereby improve nasal function [9, 11]. In addition, evidence has suggested that the regulation of neuro-endocrine-immune networks may common to acupuncture treatments of AR [12, 13]. The aim of this study was to assess the effectiveness and safety of treating PAR with IA and assess the feasibility of conducting clinical trials on a larger scale.

## Methods/design

### Ethical statement

The study was conducted in accordance with the Declaration of Helsinki Good Clinical Practice guidelines. It was approved by the Institutional Review Boards of Beijing University of Chinese Medicine (approval number BZYYYDX-LL-20150208), which is a central ethics committee providing approval for our center. Written informed consent was acquired from all participants.

### Trial design

**Fig. 2:**
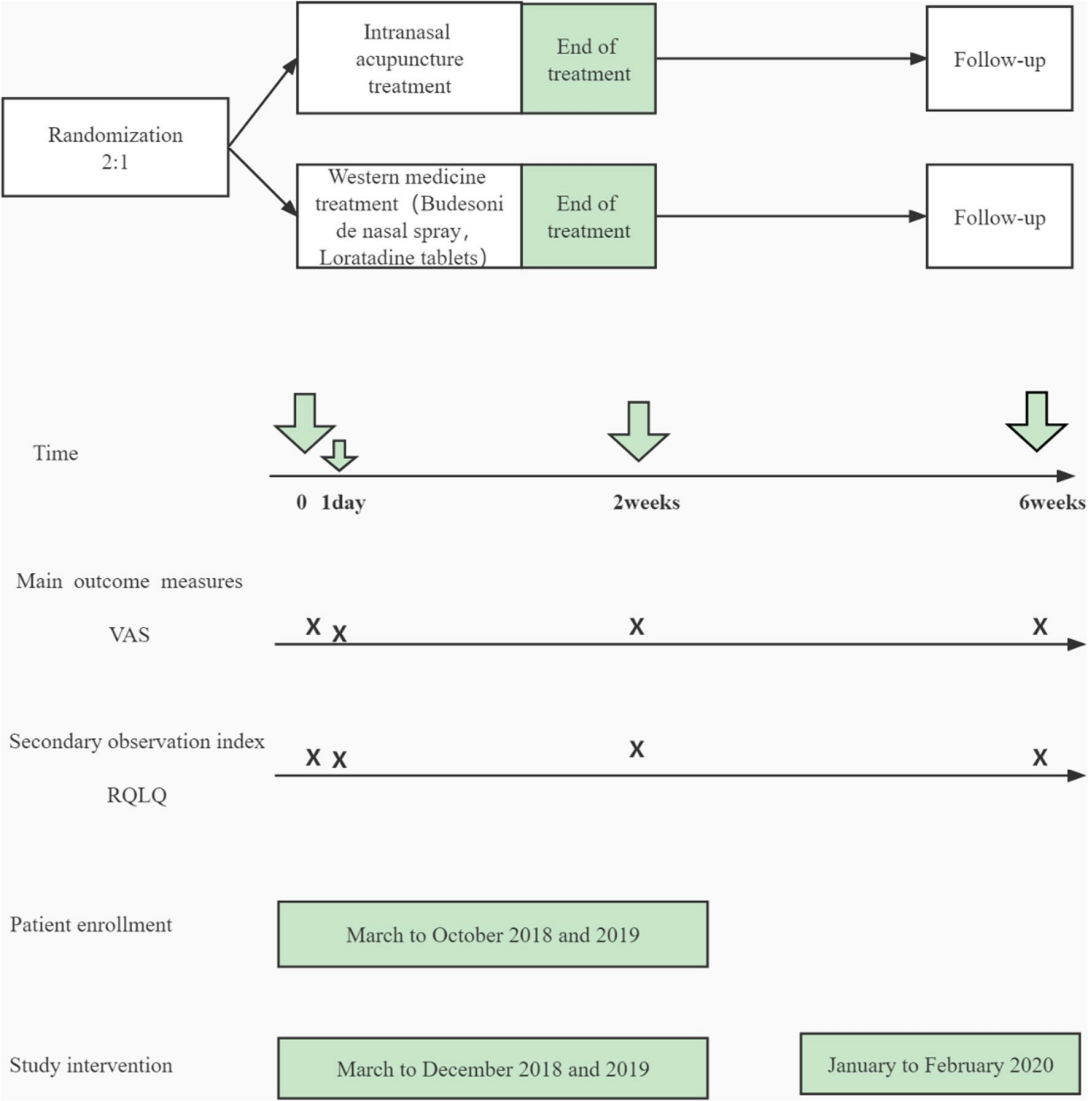
Study design. *IAG* intranasal acupuncture group, *WMG* Western medicine group, *VAS* visual analog scale, *RQLQ* Rhinoconjunctivitis Quality-of-Life Questionnaire

In the clinical pre experiment of the research group, the nasal symptoms (especially nasal congestion) of patients were significantly improved after acupuncture for 3 times. When acupuncture for 7 times, the curative effect reached a stable stage. We designed a single center trial that randomly assigned patients to 2 weeks of IA or Western medicine treatment, and we assessed visual analog scale (VAS) scores as main outcomes and Rhinoconjunctivitis Quality-of-Life Questionnaire (RQLQ) scores as secondary measurements at the end of the first treatment (1 day), at the end of treatment (2 weeks), and 4 weeks after treatment (at 6 weeks) (Fig. 2).

The study was conducted at the Department of Ear, Nose and Throat (ENT) in the Dongzhimen Hospital Beijing University of Chinese Medicine in China. In this study, the patients were guided to stop taking anti-allergy medications for at least 7 days. After completing the baseline assessment, the patients were randomized into either the intranasal acupuncture group (IAG) or Western medicine group (WMG) and received 2 weeks of IA treatment (because intranasal acupuncture has a direct stimulative effect on the nasal mucosa, it needs self-healing of the mucosa after acupuncture. Acupuncture is selected every other day for a total of 7 times, so the treatment course are 2 weeks) or budesonide nasal spray combined with loratadine tablets (Western medicine). The patients completed all primary and secondary measures at baseline, 2 weeks and 6 weeks. Adverse events were identified during intervention sessions and follow-up interviews.

### Participants

The eligible participants were older than 18 years and met the criteria of moderate to severe PAR, according to the criteria listed in “Allergic Rhinitis and its Impact on Asthma (ARIA)” [14].

The inclusion criteria included symptoms that had persisted for more than 4 days per week for more than four consecutive weeks and at least one of the following rhinitis-associated conditions: nasal obstruction, rhinorrhea, sneezing, and nasal itching. All included participants exhibited at least one positive result on an allergy skin prick reaction test at screening. All participants had a certain degree of drug resistance to glucocorticoids in the past. The participants were excluded if they suffered from serious medical conditions, such as uncontrolled hypertension, insulin-dependent diabetes mellitus, past or current malignant tumor, severe dyslipidemia, liver or kidney dysfunction, anemia, active pulmonary tuberculosis, or other infectious or systemic diseases that would make treatment with acupuncture inappropriate. Participants were deemed ineligible if they suffered from congenital nasal abnormalities, including nasal dermoid cysts and congenital midline nasal masses, sinusitis, or asthma or deviation of nasal septum; had a history of nose surgery; had received CAM therapy for AR within the previous 6 months; or had received systemically administered corticosteroids, antihistamines, or decongestants within 6 months prior to enrollment. During acupuncture, we usually use nasal endoscopy for auxiliary treatment, and do not use local anesthesia spray to promote needle insertion.

### Randomization and interventions

A total of 120 eligible patients were recruited and were randomly assigned at a 2:1 ratio to the IAG or WMG. Randomization sequences were generated using SAS software (SAS Institute Inc., Cary, NC, USA). Random numbers were concealed using sequentially numbered, opaque, sealed envelopes by an independent researcher who was not involved in the clinical trial. The doctors and participants in the IAG and WMG could not be blinded to the treatment assignments given the nature of the interventions. Outcome assessors, data collectors, and statisticians were blinded to the treatment allocation. Due to the time limit of recruitment (March 2018 December 2019), patients with PAR mostly seek a variety of traditional Chinese medicine alternative treatment, and there are fewer patients treated with intranasal acupuncture alone or Western medicine alone, resulting in a relatively insufficient sample size.

We developed the trial interventions in a consensus process with experienced acupuncture experts. In the IAG, participants received 7 treatments, that is, once every other day for 2 weeks. The acupuncture sites for IA treatment were selected to be Neiyingxiang (EX-HN9) and Biqiu points in the bilateral nasal cavity (Fig. 1). The applicator held a nasal endoscope in his left hand and a 0.30 × 75 mm filiform needle in his right hand. When aiming at the position (Neiyingxiang point and Biqiu point), the applicator quickly inserted the needle to a 20-mm depth and parallel to the inferior turbinate or middle turbinate, without special reinforcing and reducing techniques. The acupuncture stimulation lasted for 20 min in each treatment session.

**Fig. 3:**
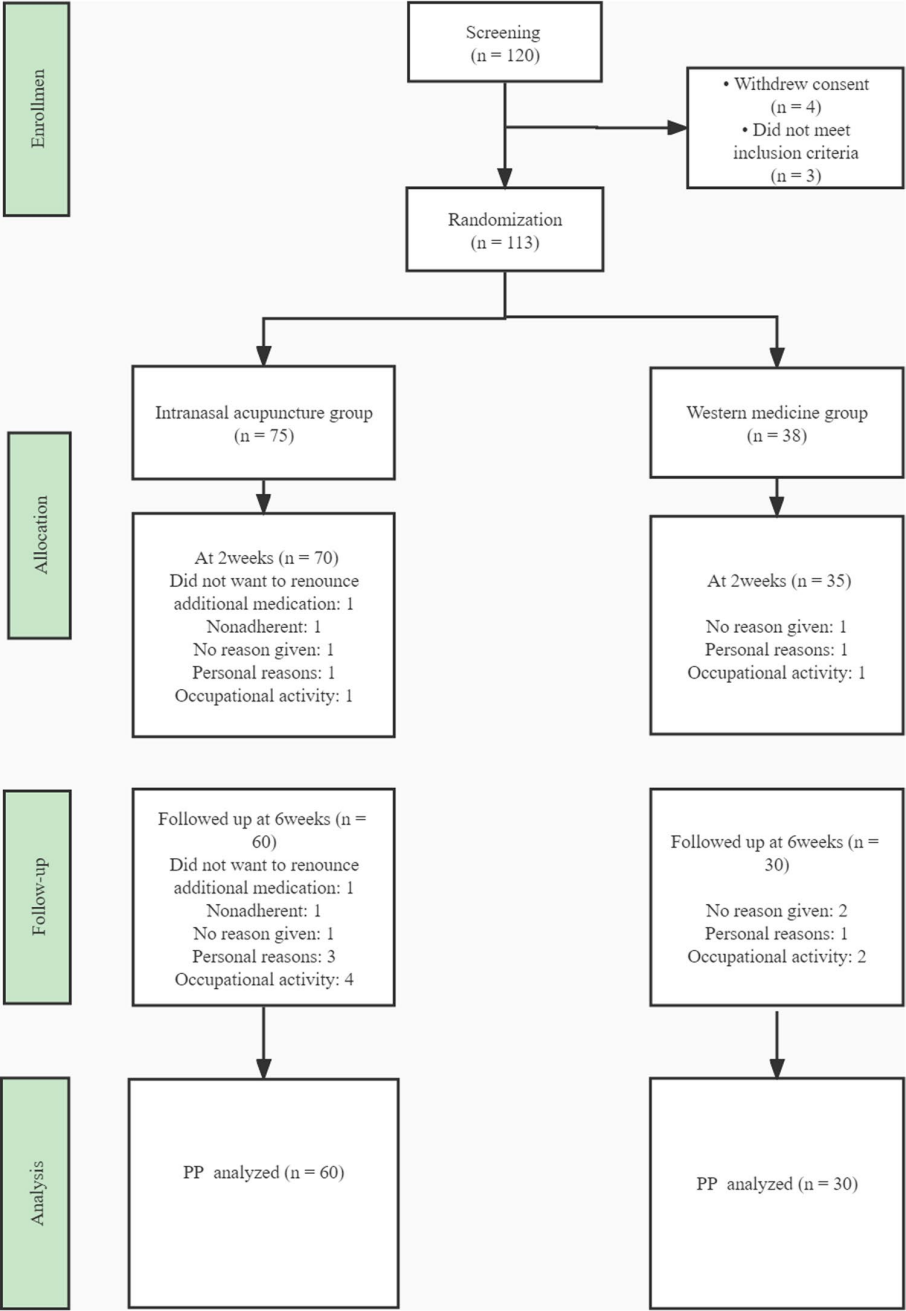
Study flow diagram. *IAG* intranasal acupuncture group, *WMG* Western medicine group, *VAS* visual analog scale, *RQLQ* Rhinoconjunctivitis Quality-of-Life Questionnaire, *PP* per-protocol

According to the guidelines for the treatment of AR recommended by ARIA in 2016 [2], the participants in the WMG received 2 weeks of intranasal corticosteroids and H1-antihistamines. For the WMG, budesonide nasal spray (twice a day, two spray for each nostril; Astra Zeneca AB, Sweden) and loratadine tablets (one tablet per night; Bayer Pharmaceutical (Shanghai) Co., Ltd.). Both groups were followed up for assessment at the end of treatment and 4 weeks later (Fig. 3).

### Outcome measurements

The primary outcomes were VAS scores for nasal symptoms. The scale includes overall nasal symptoms and differentiated nasal symptoms. The highest score for each section is 10, which represents a symptom that is unbearable; the lowest score for each section is 0, which represents a symptom with no influence.

Secondary outcomes were measures of participant quality of life, which were assessed with the RQLQ [15]. We assessed symptoms using the RQLQ, which has 28 questions covering 7 domains (activity limitations, sleep problems, nose symptoms, eye symptoms, other symptoms, practical problems, and emotional function) ranked from 0 (no impairment) to 6 (severe impairment) [16]. In addition, any adverse events were also recorded during the study period.

### Statistical analysis

The statistical analysis was performed using the SPSS statistical software system (SPSS Inc., Chicago, IL; version 22.0). Data are represented as means and standard deviations. Nonparametric tests were used for comparisons across groups for pretreatment and posttreatment data that were not normally distributed. Intragroup comparisons were performed using paired t tests or Wilcoxon rank-sum tests. Two-tailed *P* values < 0.05 were considered statistically significant.

## Results

From March 2018 through October 2019, 120 patients seen in the Department of Otolaryngology of Dongzhimen Hospital of Beijing University of Traditional Chinese Medicine were randomly selected. Three people who did not meet the acceptance and discharge standards and four who did not sign informed consent were excluded. A total of 113 patients were randomly divided into two groups: 75 in the IAG (40 women and 35 men) and 38 in the WMG (18 women and 20 men). After the treatment, 70 people (37 women and 33 men) remained in the IAG (1 patient did not want to give up additional drug treatment; 1 was noncompliant; 1 did not provide a reason; 1 left for personal reasons; 1 left for professional activities), and 35 people (17 women and 18 men) remained in the WMG (1 patient did not provide a reason; 1 left for personal reasons; 1 left for professional activities). During the 1-month follow-up period after the end of treatment, there were 60 people (31 women and 29 men) in the IAG (1 patients did not want to give up additional drug treatment; 1 was noncompliant; 1 did not provide a reason; 3 left for personal reasons; 4 left for professional activities) and 30 people (15 women and 15 men) in the WMG (2 patients did not give reasons; 1 left for personal reasons; 2 left for professional activities). Finally, 60 people were enrolled in the IAG and 30 in the WMG (Fig. 3; Table 1).

**Table 1:** Comparison of participant characteristics in the two groups

Characteristic	Intranasal acupuncture group	Western medicine group
Male, *n* (%)	31 (51.7%)	15 (50%)
Female, *n* (%)	29 (48.3%)	15 (50%)
Mean age (SD), y	40.98 ± 10.35	41.46 ± 10.99
History of PAR	8.48 ± 3.20	7.26 ± 3.01
Basic mean VAS score (SD)	44.08 ± 8.689	39.23 ± 9.038
Basic mean RQLQ score (SD)	107.35 ± 23.59	96.51 ± 19.71

Expressed as means ± SDs unless otherwise stated

*PAR* persistent allergic rhinitis, *VAS* visual analog scale, *RQLQ* Rhinoconjunctivitis Quality-of-Life Questionnaire

*P* > 0.05 indicates that there was no statistically significant difference

**Fig. 4:**
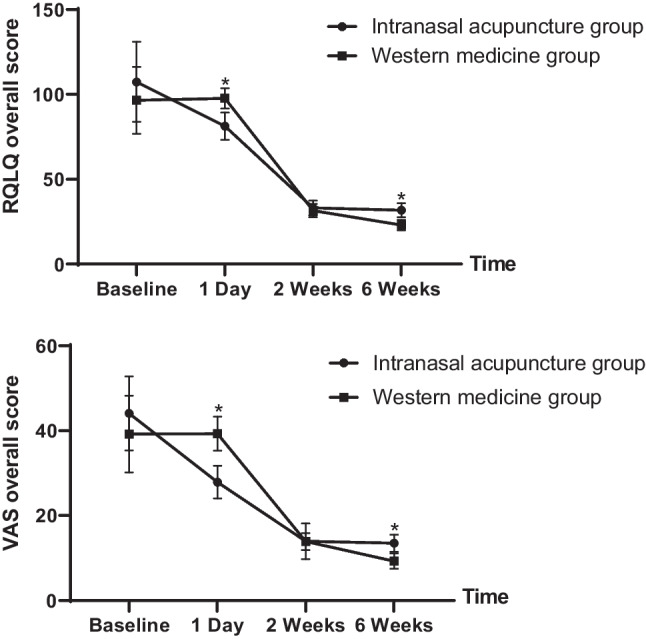
Changes in VAS and RQLQ scores in the IAG and WMG at four timepoints. Asterisks indicate the timepoints with significant differences between the two groups (*: *P* < 0.05). *IAG* intranasal acupuncture group, *WMG* Western medicine group, *VAS* visual analog scale, *RQLQ* Rhinoconjunctivitis Quality-of-Life Questionnaire

Overall, baseline characteristics were similar between the 2 study groups. After the first IA treatment, there was a significant difference in VAS scores between the IAG and the WMG (*P* < 0.05; 95% CI − 13.1 to − 9.6 points) (Table 2; Fig. 4). The scores for overall symptoms (95% CI − 2.86 to − 1.86 points), nasal obstruction (95% CI − 6.33 to -5.36 points) and olfactory function (95% CI − 2.91 to − 1.75 points) were significantly improved (Table 3). After the end of treatment, VAS scores in the two groups decreased; however, scores in the two groups were not significantly different at week 2 (*P* > 0.05; 95% CI − 1.21 to − 1.38 points) (Table 2). The olfactory function symptoms were significantly improved (95% CI − 1.58 to − 0.21 points) (Table 3). After the 1-month follow-up period after the end of treatment, there was a significant difference between the two groups (*P* < 0.05; 95% CI 2.39 to 4.1 points) (Table 2), but the VAS scores were higher in the IAG than in the WMG. The symptoms of rhinobyon, nasal itch, rhinorrhea and olfactory function were significantly improved (Table 3).

**Table 2:** VAS scores and RQLQ scores between the two groups (mean ± SD)

Outcome	Intranasal acupuncture group	Western medicine group	*P* value	Mean change from baseline (95% CI)	
VAS					
Baseline	44.08 ± 8.689	39.23 ± 9.038	0.12	– 0.63	5.3
1 day	27.9 ± 3.80	39.3 ± 4.02	0.01	– 13.1	– 9.6
2 weeks	13.98 ± 4.20	13.9 ± 1.98	0.89	– 1.21	1.38
6 weeks	13.51 ± 1.99	10.26 ± 1.79	0	2.39	4.1
RQLQ					
Baseline	107.35 ± 23.59	96.51 ± 19.71	0.18	– 2.36	12.06
1 day	81.35 ± 10.66	97.63 ± 7.984	0	– 20.27	– 12.28
2 weeks	33.16 ± 4.41	31.6 ± 3.97	0.1	– 0.33	3.46
6 weeks	31.81 ± 4.18	25.63 ± 3.54	0	4.41	7.95

*VAS* visual analog scale, *RQLQ* Rhinoconjunctivitis Quality-of-Life Questionnaire

*P* values are for comparison of values in the IAG and WMG

**Table 3:** VAS scores for overall symptoms, rhinobyon, nasal itch, sneezing, rhinorrhea and olfactory function between the two groups (mean ± SD)

Outcome	Intranasal acupuncture group (*n* = 95)	Western medicine group	*P* value	Mean change from baseline (95% CI)	
Overall symptoms					
Baseline	8.13 ± 1.43	7.93 ± 1.17	0.51	– 0.4	– 0.8
1 day	5.1 ± 1.20	7.46 ± 1.07	0	– 2.86	– 1.86
2 weeks	2.55 ± 1.18	2.23 ± 1.22	0.24	– 0.22	0.85
6 weeks	2.56 ± 0.94	1.9 ± 0.75	0.001	0.29	1.03
Rhinobyon					
Baseline	7.88 ± 1.67	7.6 ± 0.96	0.31	– 0.27	0.83
1 day	1.31 ± 0.56	7.16 ± 1.23	0	– 6.33	– 5.36
2 weeks	2.35 ± 1.07	2.03 ± 0.88	0.16	– 0.13	0.76
6 weeks	2 ± 0.78	1.6 ± 0.72	0.21	0.06	0.73
Nasal itch					
Baseline	7.11 ± 2.27	6.73 ± 1.50	0.34	– 0.41	1.18
1 day	6 ± 1.37	6.4 ± 1.45	0.2	– 1.02	0.22
2 weeks	2.1 ± 1.08	2.03 ± 0.76	0.73	– 0.32	0.46
6 weeks	1.93 ± 0.84	1.5 ± 0.68	0.016	0.08	0.78
Sneeze					
Baseline	7.7 ± 1.88	7.13 ± 1.45	0.28	– 0.38	1.28
1 day	6.11 ± 1.35	6.46 ± 1.07	0.74	– 0.69	0.49
2 weeks	2.41 ± 1.04	2.33 ± 0.88	0.51	– 0.41	0.81
6 weeks	2.78 ± 1.05	1.93 ± 0.63	0.216	– 0.15	0.69
Rhinorrhea					
Baseline	7.75 ± 2.07	7.3 ± 1.39	0.28	– 0.38	1.28
1 day	6.16 ± 1.39	6.26 ± 1.25	0.74	– 0.69	0.49
2 weeks	2.63 ± 1.40	2.43 ± 1.33	0.51	– 0.41	0.81
6 weeks	2.06 ± 1.00	1.8 ± 0.84	0.52	– 0.15	0.69
Olfactory function					
Baseline	5.5 ± 3.44	5.03 ± 2.05	0.45	– 0.69	1.62
1 day	3.23 ± 1.31	5.56 ± 1.27	0	– 2.91	– 1.75
2 weeks	1.93 ± 1.68	2.83 ± 1.20	0.01	– 1.58	– 0.21
6 weeks	2.16 ± 0.76	1.53 ± 0.62	0	0.31	0.95

*VAS* visual analog scale

*P* values are for the comparison of values in the IAG and WMG

After the first IA treatment, RQLQ scores in the IAG were significantly different from those in the WMG (*P* < 0.05; 95% CI − 20.27 to − 12.28 points) (Table 2; Fig. 4). On the RQLQ, sleep (95% CI − 5.05 to − 3.57 points), actual problems (95% CI − 2.03 to − 0.06 points), nasal symptoms (95% CI − 6.62 to − 4.5 points) and emotional problems (95% CI − 5.05 to − 3.5 points) significantly improved (Table 4). After the end of treatment, RQLQ scores in the two groups decreased; however, scores in the two groups were not significantly different at week 2 (*P* > 0.05; 95% CI − 0.33 to -3.46 points) (Table 2). After the 1-month follow-up period after the end of treatment, there was a significant difference between the two groups (*P* < 0.05; 95% CI 4.41–7.95 points) (Table 2), but the RQLQ scores were higher in the IAG in the WMG. The symptoms related to activities, non-nasal/eye symptoms, actual problems, nasal symptoms, and eye symptoms were significantly improved (Table 4).

**Table 4:** RQLQ scores for activities, sleep, non-nasal/eye symptoms, practical problems, nasal problems, eye symptoms and emotional function between the two groups (mean ± SD)

Outcome	Intranasal acupuncture group (*n* = 95)	Western medicine group	*P* value	Mean change from baseline (95% CI)	
Activities					
Baseline	12.4 ± 2.76	11.5 ± 1.65	0.54	– 0.01	1.84
1 day	10.1 ± 1.91	10.9 ± 2.22	0.08	– 1.69	0.09
2 weeks	4.83 ± 1.25	4.86 ± 1.67	0.92	– 0.72	0.66
6 weeks	4.15 ± 1.05	3.06 ± 0.90	0	0.63	1.53
Sleep					
Baseline	11.2 ± 2.45	12.1 ± 1.97	0.29	– 0.78	2.54
1 day	8.58 ± 3.37	11.8 ± 2.33	0	– 5.05	– 3.57
2 weeks	3.51 ± 1.40	3.03 ± 0.92	0.57	– 0.38	0.68
6 weeks	2.43 ± 1.24	2.5 ± 0.93	0.087	– 0.04	0.68
Non-nasal/eye					
Baseline	24.7 ± 8.93	23.6 ± 3.99	0.39	– 1.54	3.87
1 day	21.2 ± 4.53	21.8 ± 2.80	0.468	– 2.11	0.97
2 weeks	8.6 ± 1.87	8.23 ± 1.99	0.39	– 0.48	1.21
6 weeks	8.65 ± 1.70	7.63 ± 1.27	0.005	0.31	1.71
Practical problems					
Baseline	14.2 ± 2.64	13.5 ± 2.67	0.24	– 0.49	1.89
1 day	11.5 ± 2.29	12.5 ± 2.04	0.03	– 2.03	– 0.06
2 weeks	4.56 ± 1.41	4.3 ± 1.11	0.33	– 0.28	0.81
6 weeks	5.3 ± 1.81	3.4 ± 1.13	0	1.27	2.52
Nasal problems					
Baseline	17.2 ± 3.62	16.1 ± 3.43	0.17	– 0.48	2.64
1 day	10.2 ± 1.82	15.7 ± 2.56	0	– 6.62	– 4.5
2 weeks	4.9 ± 1.64	5.1 ± 1.78	0.59	– 0.95	0.55
6 weeks	5.26 ± 2.03	4.33 ± 1.21	0.008	0.25	1.61
Eye symptoms					
Baseline	13.8 ± 4.91	12.8 ± 3.02	0.24	– 0.68	2.65
1 day	11.0 ± 4.30	11.7 ± 2.34	0.32	– 2.09	0.69
2 weeks	3.26 ± 2.01	2.73 ± 0.94	0.09	– 0.08	1.15
6 weeks	3.63 ± 1.66	2.63 ± 1.18	0.004	0.32	1.67
Emotional function					
Baseline	13.6 ± 5.02	12.7 ± 2.89	0.22	– 0.28	1.18
1 day	8.58 ± 1.81	12.9 ± 1.32	0	– 5.05	– 3.5
2 weeks	3.48 ± 1.33	3.33 ± 1.12	0.59	– 0.41	0.71
6 weeks	2.38 ± 1.02	2.06 ± 0.69	0.087	– 0.04	0.68

*RQLQ* Rhinoconjunctivitis Quality-of-Life Questionnaire

*P* values are for the comparison of values in the IAG and WMG

During the clinical trial, the patients in the IAG experienced a small amount of bleeding (< 2 ml). The blood can be stopped by tamping the nostril for 20 min. No other adverse events, such as local hematoma, were reported in either group during the clinical trial. In addition, no patient suspended the treatment due to pain.

## Discussion

Glucocorticoid resistance [17, 18] is a common cause of moderate to severe AR, and also a difficult point in clinical treatment. The patients included in this study are perennial moderate to severe allergic rhinitis, with a long history, certain resistance to intranasal steroids. In this randomized trial of IA for PAR, compared with medication (1 day of antihistamine use), IA (with the first treatment) led to improvements (nasal obstruction and olfactory function) on the VAS and with sleep, actual problems, nasal symptoms and emotional problems related to disease-specific quality of life. However, there was no significant difference in the efficacy of nasal acupuncture compared with Western medicine in the responses at 2 weeks. After the 1-month follow-up period after the end of treatment, the effects observed in the IAG decreased compared with that in the WMG. The results showed that with the extension of the time past the end of treatment, the effect of IA may decrease, although the immediate effects were obvious. IA can directly treat the nasal mucosa in a particular area rich in nerve blood vessels (e.g., in AR), and IA can rapidly improve the pathological changes in the nasal mucosa. The strong effects of IA can be adjusted and are more direct and rapid than conventional acupuncture; compared with acupuncture *Sphenopalatine ganglion *(*SPG*) [11] and GeZhi nasal hillock special treatments, IA also has many characteristics, such as direct effects on disease targets, ease of operation and high safety, which could lead to its promotion. This study was the first to evaluate the efficacy of IA in the treatment of AR in a controlled clinical trials.

Our previous research found that nasal acupuncture addresses AR-related nasal mucosal and immune system interactions and the Th1/Th2 imbalance, such that Th2 cells and the secretion of cytokines promotes a Th1/Th2 balance. We also found that clinical symptoms following nasal acupuncture improved, and there was regulation of serum eosinophils and cell counts of IL-4 in nasal secretions in patients with AR [19]. INF-gamma and SP neuropeptides have a role in future projects that will further characterize the mechanisms of nasal acupuncture in relation to Th1/Th2 cytokines and further explore neurogenic inflammation. At the same time, through the study of the functional magnetic resonance imaging (fMRI) of patients with nasal acupuncture, it [20] was found that nasal acupuncture can be adjusted to a certain extent through the nasal ventilation volume; the parahippocampal gyrus, amygdala, hippocampal lobe, and insula were clearly stimulated such that we speculated that nasal acupuncture was a special kind of needle acupuncture method. Evaluation of the regulation of the central nervous system–immunoreactive networks will provide further evidence [21].

Acupuncture effects produced by stimulation of the corresponding meridian acupoints, and through these meridians that are adjusted through the skin, functional adjustments can be made, the balance of Yin and Yang to correct the pathological phenomena of partial tide can occur, and the function of the body can be brought back to normal status. To achieve the purpose of treating diseases, research has shown that acupuncture points may be a low resistance point, i.e., a subcutaneous adjustment system (meridian) and hub. Acupuncture may be one method by which the central nervous system can be stimulated; subsequent adjustments to the neuroendocrine and humoral immune networks in the central nervous system can then, in turn, affect the target organs. fMRI can monitor the activity of specific brain functional areas under conditions of acupuncture that extract qi. Visualization of the activity of specific acupuncture effects in the brain may provide an effective means for exploring the mechanisms of acupuncture based on the theory of TCM coupled with medical imaging technology.

In the current clinic, we have found [22] that when the intranasal acupuncture treatment for AR patients is extended to 4 weeks, the effect will be more durable and repeatable, which lacks large-scale clinical data support. In view of the long-term effect of intranasal acupuncture, our research group is further discussing the design. After each acupuncture and 2 weeks of treatment, we will observe how long the patient's symptoms will last. We will continue the acupuncture treatment for 2 weeks to observe whether the effect of intranasal acupuncture will last.

## Conclusions

Nasal acupuncture is an effective treatment for PAR, as it may ameliorate nasal symptoms, improve quality of life, and reduce the dosage of medicine. In conclusion, this study proved that nasal acupuncture is an effective and reliable method for the clinical treatment of PAR with a stable curative effect and is worthy of promotion in clinical application, although the long-term curative effects and mechanisms of action remain to be further studied. Therefore, further validation of these results with larger scale, multicenter clinical trials using more objective indicators could be decisive.

## Data Availability

The datasets used/analyzed during the current study are available from the corresponding author on reasonable request.

## Abbreviations

AR: Allergic rhinitis
CAM: Complementary and alternative medicine
ENT: Ear, nose and throat
IL: Interleukin
ICFs: Informed consent forms
PAR: Persistent allergic rhinitis
IAG: Intranasal acupuncture group
WMG: Western medicine group
VAS: Visual analog scale
RQLQ: Rhinoconjunctivitis Quality-of-Life Questionnaire
SPA: Sphenopalatine acupoint
SPG: Sphenopalatine ganglion
